# Evaluation of Handwriting Movement Kinematics: From an Ecological to a Magnetic Resonance Environment

**DOI:** 10.3389/fnhum.2016.00488

**Published:** 2016-09-29

**Authors:** Ambra Bisio, Ludovico Pedullà, Laura Bonzano, Piero Ruggeri, Giampaolo Brichetto, Marco Bove

**Affiliations:** ^1^Department of Experimental Medicine, Section of Human Physiology and Centro Polifunzionale di Scienze Motorie, University of GenoaGenoa, Italy; ^2^Department of Neuroscience, Rehabilitation, Ophthalmology, Genetics, Maternal and Child Health, University of GenoaGenoa, Italy; ^3^Scientific Research Area, Italian Multiple Sclerosis FoundationGenoa, Italy

**Keywords:** kinematics, magnetic resonance-compatible tablet, fMRI, test–retest, ecological validity

## Abstract

Writing is a means of communication which requires complex motor, perceptual, and cognitive skills. If one of these abilities gets lost following traumatic events or due to neurological diseases, handwriting could deteriorate. Occupational therapy practitioners provide rehabilitation services for people with impaired handwriting. However, to determine the effectiveness of handwriting interventions no studies assessed whether the proposed treatments improved the kinematics of writing movement or had an effect at the level of the central nervous system. There is need to find new quantitative methodologies able to describe the behavioral and the neural outcomes of the rehabilitative interventions for handwriting. In the present study we proposed a combined approach that allowed evaluating the kinematic parameters of handwriting movements, acquired by means of a magnetic resonance-compatible tablet, and their neural correlates obtained simultaneously from a functional magnetic resonance imaging examination. Results showed that the system was reliable in term of reproducibility of the kinematic data during a test/re-test procedure. Further, despite the modifications with respect to an ecological writing movement condition, the kinematic parameters acquired inside the MR-environment were descriptive of individuals’ movement features. At last, the imaging protocol succeeded to show the activation of the cerebral regions associated with the production of writing movement in healthy people. From these findings, this methodology seems to be promising to evaluate the handwriting movement deficits and the potential alterations in the neural activity in those individuals who have handwriting difficulties. Finally, it would provide a mean to quantitatively assess the effect of a rehabilitative treatment.

## Introduction

Handwriting is historically believed to be one of the most difficult fine motor skills to learn. It takes years of practice before a person has mastered the mature handwriting skill. Despite the difficulties this ability can pose to the learners, handwriting is one of the first motor expertise that children acquire at school and is nowadays part of the individual motor-cultural baggage. Although the increasing use of personal computer has reduced the time dedicated to handwriting, adults still use handwriting to communicate with others, to record ideas and for creative expression.

Handwriting is a complex functional activity, which involves fine motor skills, cognitive and visual-perceptual processing (Mary-Ann, 1992; Feder and Majnemer, 2007). In adults, the ability to handwrite can be affected, or even lost, due to neurological diseases. For instance, handwriting deficits are common after stroke (Simpson et al., 2015) and in people with multiple sclerosis (Wellingham-Jones, 1991; Schenk et al., 2000), Parkinson’s disease (Van Gemmert et al., 1999, 2001; Lange et al., 2006), and obsessive-compulsive disorders (Mavrogiorgou et al., 2001).

The restoration of handwriting is matter of the occupational therapy treatments. Unfortunately, many of the conventional methods used to evaluate handwriting movements during the clinical routine are not standardized and are dependent on the therapist’s expertise (Hoy et al., 2011; Van Drempt et al., 2011). From two recent reviews on retraining methodologies for handwriting ability (Hoy et al., 2011; Yancosek and Howell, 2011) emerged that no studies assessed the effect of the proposed treatment on the kinematics of writing movement or on the central nervous system activity. Therefore, it would of great importance to quantitatively describe handwriting motor performance and to investigate brain activity during handwriting. The application of this combined methodology could also allow assessing possible changes in the kinematics and in the neural activations after a rehabilitation treatment. This is one of the reasons why new digital approaches, which make use of technology for data collection and kinematic evaluation, have been developed. To this concern, recent studies on handwriting movements were carried out by means of digitizing tablets that allow a quantitative analysis of the kinematic parameters of the writing trace (Mergl et al., 1999; Van Gemmert et al., 1999, 2001; Schenk et al., 2000; Mavrogiorgou et al., 2001; Vuillermot et al., 2009; Accardo et al., 2013). Other studies investigated handwriting by using algorithms able to translate electromiographic signals generated by hand and forearm muscles into handwriting traces (Linderman et al., 2009; Okorokova et al., 2015).

A step further toward a comprehensive description of the laws that govern handwriting is the acquisition of the neural correlates of writing movements with the aim to investigate the relationship between the behavioral data and the cerebral activity. In this context, functional magnetic resonance imaging (fMRI) has become a powerful tool to study the functional organization of the brain (Menon, 2001; Savoy, 2001), allowing the acquisition of brain activation during the motor task. Thus, it is also useful to develop MR-compatible devices to perform a kinematic recording of the writing movement during fMRI examination, even though it poses a problem related to the validity of the kinematic data because of the postural constraints the subject is forced to maintain in the MR scanner. Until now some efforts have been done in this direction, but only few studies tackled this issue (Katanoda et al., 2001; Siebner et al., 2002; Reithler et al., 2006; Reitz et al., 2013; Karimpoor et al., 2015) and none of them tested whether the kinematic features of the subject’s movements obtained in ecological writing conditions (i.e., when the subject is seated at a table) are preserved when the task is performed inside the MR scanner. Indeed, one might hypothesize that when a person moves inside the narrow space of the MR environment the handwriting movement performance changes. Furthermore, among these studies, only Karimpoor et al., 2015 provided a simultaneous visual feedback of the subject’s performance showing a reconstruction of the participant’s hand during handwriting together with the written trace. Although this system was demonstrated to improve the quality of the kinematic measurements with respect to a non-visual feedback condition, this kind of visual feedback could also be distracting because the subject’s focus may move from the recognition of the hand to the written trace or also other details of the displayed image.

Aim of the present work was to implement a methodology able to quantitatively characterize the kinematics of handwriting movements and the related neural substrates. Indeed, in this study we considered the temporal and spatial patterns of handwriting, omitting the evaluation of writing *per se*. To achieve this goal we: (a) assessed the reproducibility of repeated measurements of handwriting movements acquired by means of a newly designed and developed MR-compatible tablet outside the magnet bore, (b) tested the reliability of the kinematic data acquired inside the MR scanner, where participants had a visual simultaneous visual feedback of their written trace, by comparing them with those obtained in ecological condition, and (c) investigated the neural correlates associated to handwriting by means of fMRI sequences.

## Materials and Methods

This study was composed of two experiments: Experiment 1 assessed the reproducibility of the kinematic data acquired with the MR-compatible tablet by means of a test–retest approach. Since this was the first study that used this system (i.e., the combination of this MR-compatible tablet with our custom-made acquisition software) and this methodology, it was crucial to test its reliability. Experiment 2 tested whether the individual kinematic features associated with handwriting movements performed in ecological conditions are maintained when participants performed the task inside the MR scanner. Further, in a sub-group of subjects, who took part to Experiment 2, we tested whether the proposed kinematic paradigm was able to evoke the activation of the cerebral areas usually active during a handwriting task.

### Participants

A total of 44 subjects were enrolled in this study. Twenty-two of them (12 females and 10 males, mean age ± SD = 25.0 ± 5.6 years) participated in Experiment 1, whereas 22 (14 females and 8 males, mean age ± SD = 24.2 ± 6.1 years) took part in Experiment 2. Seven out of the 22 subjects who took part to Experiment 2 were also recruited for an fMRI examination. Participants who manifested MR contraindications, as claustrophobia, pregnancy, and presence of a pacemaker and other metallic parts not magnetic resonance compatible were not considered eligible for Experiment 2. All subjects were right-handed according to the Edinburgh Handedness Inventory (Oldfield, 1971) and naive to the specific purpose of this study. People with neurological disturbances were excluded from the study. Informed consent was obtained according to a procedure approved by the local ethics committee (Comitato Etico Regione Liguria, IRCCS Azienda Ospedaliera Universitaria San Martino—IST, Genoa, Italy) and to the Declaration of Helsinki.

### Equipment for Handwriting Movement Acquisition

#### Hardware

**FIGURE 1:**
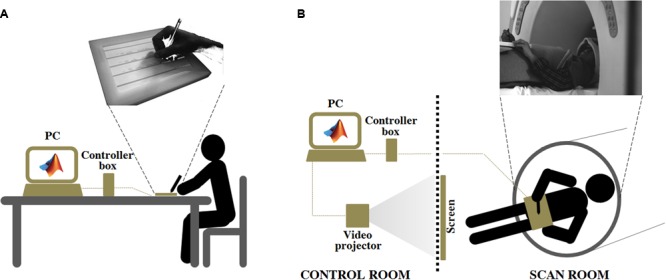
**Experiment 2: Experimental set up adopted for the ecological handwriting movement condition, outside **(A)**, and inside **(B)** the magnetic resonance environment.** In the condition OUTSIDE the subjects, seated at a table, used an ink stylus to write on a paper with black, horizontal lines positioned over the SMART TAB surface. The experimenter monitored the formation of the written trace on the computer screen connected to the MR tablet through the controller box. The computer managed the acquisition by means of a custom-made MatLab^®^ software. In the condition INSIDE the subject laid down inside the magnetic bore. The MR tablet was positioned on her/his body at the level of the pelvis. A pad was placed under the tablet to help the subject to keep a position as comfortable as possible during the handwriting movement. A plastic, MR-compatible stylus was used to perform the task directly on the tablet surface. The participant had a simultaneous visual feedback of her/his motor performance by looking at a mirror positioned on the head coil. The mirror reflected the images projected onto a white screen placed inside the scan room, where a video-projector, connected to the computer used for the acquisition, displayed the written trace. The video-projector, the controller box and the computer were placed in the control room.

An innovative MR-compatible tablet, the SMART TAB (E.M.S., S.r.l., Bologna), was used to acquire handwriting movements during the evaluation sessions outside and inside the MR scanner. The SMART TAB consists of a touch-sensitive tablet, a plastic-made stylus, a USB controller box, and a cable that connects the tablet with the controller outside the magnet room. All the equipment inside the magnet room is non-ferromagnetic. The surface of the tablet is the AccuTouch Five-Wire Resistive Touchscreens (Elo Touch Solutions, Inc., Milpitas, CA, USA), where both X and Y measurements are made on a stable rear glass layer. The touch surface (spatial resolution: 4096 DPI, temporal resolution: 10 ms) is mounted within a frame that delimits the sensitive area while offering some protection from unintentional touches. The subject can use the fingers or the plastic stylus to write on the tablet (Touch Activation Force less than 113 g). The tablet is connected to the USB controller box in the operator console room via a shielded cable. In turn, the controller box is connected via USB cable to the PC that manages acquisition (**Figure 1**).

#### Software

The software handling the acquisition of writing movements was developed in our laboratory in MatLab^®^ platform, exploiting the Psychophysics Toolbox (Brainard, 1997; Kleiner et al., 2007), a free set of MatLab^®^ functions. It simultaneously provides a visual feedback of what the subject is writing on a computer monitor. When the software runs a white screen appears on the PC monitor and the software is ready for data acquisition. The data correspond to the 2D coordinates (X, Y) of the pen-tip acquired at a sampling frequency corresponding to the refresh rate of the video projector (60 Hz). In the present study the pen was either an ink stylus (when the participants wrote over a paper positioned on the tablet, see Experiment 2 - OUTSIDE condition) or a plastic made stylus without ink (when the participant wrote directly over the tablet surface, see Experiment 1, Experiment 2 - INSIDE condition). When the stylus touches the screen the PC starts to acquire the data, and a black line reproducing the written trace appears. Thus, participants can monitor in real-time what they are writing. None of the subjects reported latency between their executed movement and representation of the PC monitor.

### Experimental Procedure

#### Experiment 1

Participants were seated on a chair at a table. The SMART TAB was placed on the table and the participant could adjust its position to feel as comfortable as possible. Once a comfortable position was achieved the participants were requested to keep the orientation of the tablet and of their forearm constant. A plastic, MR-compatible stylus was used to perform the task directly on the tablet surface. Participants were required to look at the computer screen where the written trace was displayed simultaneously to the motor task. They were asked to write the Italian sentence “Il carro sale al colle” (i.e., “The wagon goes up the hill”), keeping the text in a single line. This sentence was chosen because it was short and easy for the subject to keep it in a single line, and because it was composed of simple words very popular in the Italian language. The sentence was repeated five times. Participants were tested twice (PRE and POST sessions, five repetitions of the sentence for each session), one month apart, to assess possible changes in measurements taken under the same condition and to define the motor performance parameters showing good repeatability (i.e., test–retest reliability).

#### Experiment 2

##### Handwriting movement acquisition

This experiment was composed of two sessions, which involved 22 participants. The first session (OUTSIDE) was aimed at evaluating handwriting movement outside the magnet bore, in ecological condition, i.e., participants were seated on a chair at a table. As in Experiment 1, the tablet laying on the table was positioned by the participant to feel as comfortable as possible. In addition, in order to mimic a conventional handwriting condition while recording movement kinematics, a paper with black, horizontal lines was positioned over the SMART TAB surface and the subject was provided with an ink stylus. In this way, the participant had a visual feedback over the paper of what she/he was writing and could appreciate the normal resistance of the pen over the paper during the handwriting movement; in the meantime, the tablet acquired the trajectory of the stylus and the experimenter could simultaneously monitor the writing motor output on the computer screen. The subject was asked to write three times the Italian sentence “Il sole scalda” (i.e., “The sun warms”) when a “go” signal was provided by the experimenter. As in Experiment 1, this sentence was chosen because it was composed of simple words very popular in the Italian language. We adopted a shorter sentence with respect to that of Experiment 1 in order to make the task easier for the subject inside the MR scanner.

The second session (INSIDE) took place inside the magnet bore. The subject laid down with the SMART TAB positioned on her/his body at the level of the pelvis. The forearm was positioned over an MR-compatible support made of soft material, adjustable in height to minimize the subject’s forearm movements. A pad was placed under the tablet to help the subject to keep a position as comfortable as possible during the handwriting movement. A plastic, MR-compatible stylus was used to perform the task directly on the tablet surface. The participant had a simultaneous visual feedback of her/his motor performance by looking at a mirror positioned on the head coil. The mirror reflected the images projected onto a white screen placed inside the magnet room, where a video-projector, connected to the computer used for the acquisition, displayed the written trace. The horizontal lines over the writing space were physically created by mounting equidistant, black cotton threads over the white screen inside the magnet room. The task was adapted to cover a temporal interval of 30 s, which corresponded to the active task blocks of the fMRI acquisition (performed only by seven subjects, see *Magnetic Resonance Imaging Protocol*). For this reason, participants were instructed to write the sentence on a first line and to repeat it on the lines below until a “stop” signal was provided. Each subject completed three task blocks, containing at least one complete sentence each, interleaved with 30-s periods of rest. The sentence was the same adopted for the OUTSIDE condition. During rest blocks subjects had to handle the stylus without moving and to keep the eyes opened, as in the task blocks, to avoid spurious findings related to visual activations. Each session was preceded by a familiarization phase which allowed subjects to train themselves with the SMART TAB in this non-conventional writing condition.

##### Magnetic resonance imaging protocol

Seven out of 22 subjects, who executed the two experimental sessions of the Experiment 2, during the INSIDE session were also examined by means of an fMRI test. MRI examination was performed on a 1.5 T MR system (Signa Excite HDxt, General Electric Healthcare, Waukesha, WI, USA) and included the following series covering the whole brain: axial FLAIR sequence (slice thickness = 5 mm; TR = 9002 ms; TE = 97.5 ms; inversion time = 2250 ms; flip angle = 90°; FOV = 240 mm × 240 mm; matrix = 512 × 512) to exclude incidental findings in the enrolled subjects; axial T2-weighted sequence (slice thickness = 5 mm; TR = 6300 ms; TE = 123.7 ms; FOV = 260 mm × 260 mm; matrix = 256 × 256) used as structural reference for the fMRI acquisition; T2^∗^-weighted single-shot EPI sequences (32 slices; slice thickness = 4 mm; gap = 0.5 mm; TR = 3000 ms; TE = 40 ms; FOV = 260 mm × 260 mm; matrix = 64 × 64) for fMRI. Particularly, each fMRI run included 63 brain volumes; the first 3 volumes were discarded because of non-steady magnetization. Within each run the subject performed the handwriting motor task (i.e., active task) alternatively with a rest condition (i.e., control), according to a block designed paradigm consisting of 30-sec active task periods alternating with 30-s control periods (10 brain volumes per block).

### Data Analysis

#### Data Treatment

##### Behavioral data

The kinematic parameters of handwriting movements were computed by means of a custom-made MatLab^®^ software. The software automatically detected the beginning and the end of the sentence on the basis of the module of the velocity profile (computed over the two dimensions of the tablet, x and y): the first and the last instants (in the first line, in case of multiple lines) in which the velocity was greater than zero value corresponded to the beginning and the end of the sentence, respectively. Total duration (s) (i.e., the time employed by the subject to write an entire sentence), movement length (mm), and thickness (mm) of the sentence were considered as outcome parameters. The length corresponded to the size of the segment which connects the first and the last points of the sentence. The thickness was computed as the vertical distance between the top of the highest letter and the bottom of the lowest one.

##### Neuroimaging data

SPM12 software (Wellcome Department of Imaging Neuroscience, London, UK) was used for fMRI processing (Friston et al., 1995). For each participant, the first image was used as a reference to which all the subsequent scans were realigned, and the 6 parameters describing the rigid body transformation between each source image and the reference image were used to re-sample each image to apply motion correction. Then, slice timing was applied to minimize timing-errors between slices and the functional images were normalized to the Montreal Neurological Institute (MNI) template brain image using a 12-parameter affine transformation, re-sampled to 2 mm × 2 mm × 2 mm voxels and smoothed with an 8 mm full-width at half-maximum isotropic Gaussian kernel to increase the signal-to-noise ratio.

#### Statistical Analysis

##### Experiment 1

A paired *t*-test compared the mean kinematic parameters (average computed across the five trials) acquired for each subject the first time the participant used the MR-tablet with those acquired one month later. Further, to assess the reliability of the acquisition, a linear regression model (*POST = a ^∗^ PRE + b*, where *a* and *b* refer to the slope and intercept values of the regression line, respectively) tested the relationship among the single-values of the kinematic parameters acquired during the first evaluation and after one month on the same subjects. Pearson’s correlation coefficients are provided together with the associated *p*-value, the slopes and the intercepts values of the regression lines. To be more accurate the test–retest reliability of the motor performance parameters was assessed also by the Intraclass Correlation Coefficient (ICC) and the corresponding *p-*value (Shrout and Fleiss, 1979).

##### Experiment 2

To compare participants’ motor performance inside and outside the magnetic bore, the mean kinematic parameters (average computed across the thre trials) were compared by means of paired *t*-tests. To specifically assess the reliability of the behavioral acquisitions performed inside the magnetic bore with respect to that in ecological condition (outside the magnet), a linear regression model (*OUTSIDE = a ^∗^ INSIDE + b*) tested the relationship between the kinematic parameters in the two experimental conditions. Pearson’s correlation coefficients are provided together with the slopes (*a*) and the intercepts (*b*) values of the regression lines and the associated *p-*values. As in Experiment 1, the ICC and the corresponding *p*-value were computed for each parameter.

Concerning the fMRI data, a general linear model was used to identify the voxels with task-related signal changes at the individual level. Task-related *t* contrast images were created for each subject, with a height threshold of *p* < 0.05 FWE-corrected and extent threshold arbitrarily set at *k* = 50 voxels.

Data in the text are reported as means ± SD.

## Results

### Experiment 1

**FIGURE 2:**
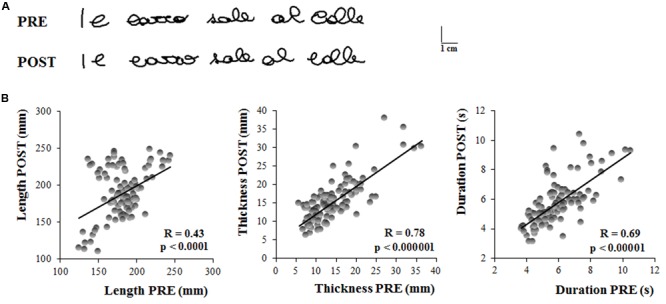
**Experiment 1. (A)** Written traces of one representative participant acquired the in first testing session (PRE) and 1 month later (POST). **(B)** These graphs show the linear regression models that describe the relationship between the kinematic parameters of the sentences written by the subjects 1 month apart. Each dot refer to a single sentence written by a participant. Each participant wrote five sentences in each testing session. Pearson’s coefficient (R) and the related level of significance (p) are reported in each graph.

**Figure 2A** shows the first sentence written by a representative subject the first time she was tested (PRE) and the first sentence she wrote 1 month later (POST). No evident differences in the sentence dimensions can be observed from these representations of the written trace.

The results of the *t*-tests showed that the length of the sentence (PRE = 183 ± 23 mm, POST = 189.47 ± 31 mm; *t* = 0.97, *p* = 0.34), as well as the thickness (PRE = 14 ± 5 mm, POST = 15 ± 4 mm; *t* = 1.71, *p* = 0.10) and the total duration of the writing movement (PRE = 6.01 ± 1 s, POST = 5.71 ± 1 s; *t* = 1.37, *p* = 0.18) did not change when retested after one month from the first acquisition.

**Figure 2B** shows the linear relationship between the kinematic parameters of each single trial acquired during the first and the second testing session (PRE and POST, respectively). Significant correlations were found between the two sessions, as shown in **Table 1**, indicating that the system (i.e., the hardware and software for the acquisition) provided reliable and repeatable data. **Table 1** reports also the ICC values for the three kinematic parameters which were found to be statistically significant. The ICC values for length and total duration were between 0.40 and 0.75, thus suggesting a fair to good reliability, whereas the ICC value for thickness was higher than 0.75, a value conventionally considered as representative of an excellent reliability (Fleiss, 1986).

**Table 1:** Description of the linear regression model and associated statistical results.

	a	b	Statistical results	ICC
**Experiment 1** *POST = a ^∗^ PRE +b*				
Length (mm)	0.57	84.34	*R* = 0.43, *p* < 0.0001	*R* = 0.42, *p* < 0.00001
Thickness (mm)	0.75	4.52	*R* = 0.78, *p* < 0.000001	*R* = 0.78, *p* < 0.00001
Duration (s)	0.74	1.33	*R* = 0.69, *p* < 0.00001	*R* = 0.69, *p* < 0.00001
**Experiment 2** *OUTSIDE = a ^∗^ INSIDE + b*				
Length (mm)	0.34	106	*R* = 0.27, *p* = 0.035	*R* = 0.75, *p* < 0.00001
Thickness (mm)	0.55	12.91	*R* = 0.35, *p* = 0.005	*R* = 0.57, *p* < 0.00001
Duration (s)	0.4	5.23	*R* = 0.35, *p* = 0.005	*R* = 0.42, *p* < 0.00001

**a* and *b* are the slope and intercept values of the regression line, respectively. In Experiment 1 the linear regressions modeled the relationship between the parameters acquired during the first testing (PRE) session and 1 month later (POST), whereas in Experiment 2 the relationship between the parameters of the sentence inside and outside the MR scanner. Pearson’s correlation coefficient (R) and the related probability are reported together with the Intrarclass Correlation Coefficient (ICC) and its *p-*value.*

### Experiment 2

**FIGURE 3:**
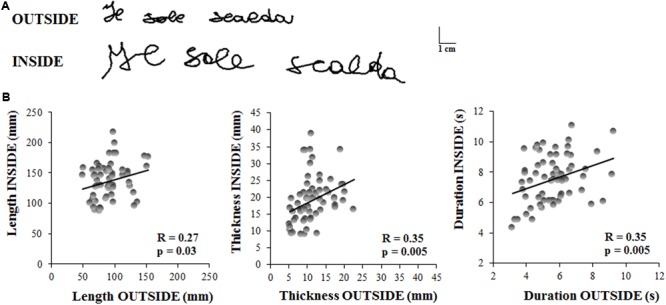
**Experiment 2. (A)** Written traces of one representative participant acquired outside and inside the magnetic resonance environment. **(B)** These graphs show the linear regression models that describe the relationship between the kinematic parameters of the sentences written by the subjects outside and inside the MR scanner. Each dot refer to a single sentence written by a participant. Each participant wrote three sentences in each condition. Pearson’s coefficient (R) and the related level of significance (p) are reported in each graph.

**Figure 3A** displays the sentence written by a representative subject outside the magnetic bore, in ecological condition, with respect to that acquired inside the magnetic bore. It is worth noting that both length and thickness of the sentence increased when the subject performed the task inside the MR scanner.

The length of the sentence was significantly longer inside than outside the MR scanner (OUTSIDE = 91 ± 23 mm, INSIDE = 137 ± 29 mm; *t* = 6.45, *p* < 0.00001). Similarly, sentence thickness increased significantly when the subjects laid down inside the scanner (OUTSIDE = 11 ± 4 mm, INSIDE = 19 ± 6 mm; *t* = 6.22, *p* < 0.00001), as well as the total duration of the writing movement (OUTSIDE = 5.65 ± 1 s, INSIDE = 7.56 ± 1 s; *t* = 5.88, *p* < 0.00001).

Despite these predictable differences between the values of the kinematic parameters acquired in ecological condition and those inside the MR scanner, we found significant correlations (see **Table 1**) between the two conditions for each parameter (**Figure 3B**), indicating that the individuals’ movement features were preserved in the MR environment. Further, the ICC values for all the kinematic parameters ranged between 0.40 and 0.75 which suggested a fair to good reliability (Fleiss, 1986).

**FIGURE 4:**
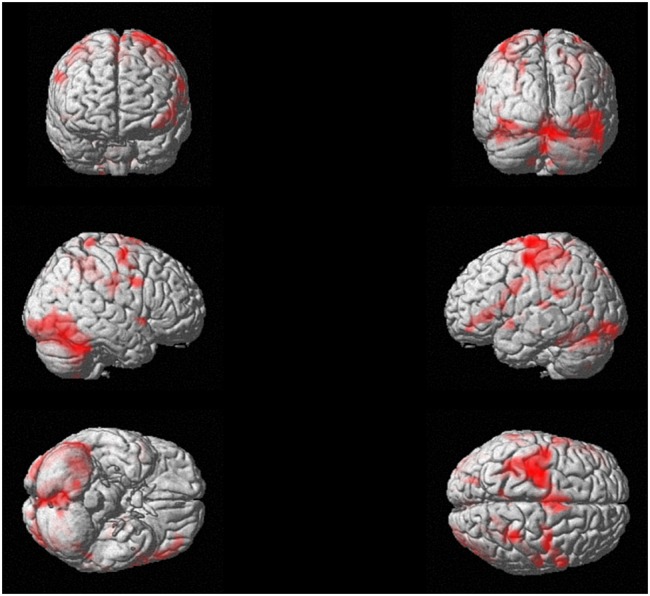
**Experiment 2.** Task-related brain activation map (*p* < 0.05 FWE-corrected; size = 50 voxels) for the handwriting movement task for a representative subject.

Concerning the analysis of fMRI data, as a first result, the healthy volunteers showed full feasibility of the procedure. In the activation maps of the single subjects, no artifactual cluster was observed. **Figure 4** shows the clusters of activation during handwriting (task vs. rest) in one representative subject on a rendered brain surface. As reported in details in **Table 2**, significant activations were found bilaterally in the frontal and parietal areas, as well as in the cerebellum. In particular, we observed significant clusters of activation with peaks in the precentral and postcentral gyri (BA 3, 4, and 6) including the left superior and medial frontal gyri. Further, the left superior parietal lobule (BA 7), and the right cerebellum were significantly activated during handwriting with respect to rest.

**Table 2:** Brain areas of activation during the handwriting task (see **Figure 4**) for a representative subject (Experiment 2).

Cluster size (voxels)	voxel T	MNI Coordinate (mm)			Hemisphere	Anatomical area	Brodmann area
		x	y	z			
5118	15.3	–26	–14	74	Left	Precentral Gyrus	6
	14.31	–46	–12	50	Left	Precentral Gyrus	4
	11.78	–40	–34	64	Left	Postcentral Gyrus	3
8806	13.93	30	–46	–32	Right	Cerebellum	
	13.08	4	–70	–12	Right	Cerebellum	
	12.81	36	–56	–30	Right	Cerebellum	
1674	12.29	58	6	32	Right	Inferior Frontal Gyrus	9
	11.85	32	–42	48	Right	Inferior Parietal Lobule	40
	9.08	54	–20	30	Right	Postcentral Gyrus	2
144	11.29	34	–40	76	Right	Postcentral Gyrus	3
847	10.19	–62	6	26	Left	Inferior Frontal Gyrus	9
	8.77	–50	32	–2	Left	Inferior Frontal Gyrus	47
	8.17	–36	48	–14	Left	Superior Frontal Gyrus	11
72	10.02	–66	–26	10	Left	Superior Temporal Gyrus	42
609	9.78	40	–10	62	Right	Precentral Gyrus	6
	7.66	48	–4	50	Right	Precentral Gyrus	6
185	9.38	–62	–40	22	Left	Superior Temporal Gyrus	22
	7	–58	–32	26	Left	Inferior Parietal Lobule	40
138	9.25	–54	8	–8	Left	Superior Temporal Gyrus	38
293	8.96	–26	–60	24	Left	Middle Temporal Gyrus	39
171	8.01	52	12	–6	Right	Superior Temporal Gyrus	22
	6.86	42	12	–6	Right	Insula	13
121	7.77	–22	–84	46	Left	Precuneus	7
	6.67	–22	–78	40	Left	Precuneus	7
86	7.66	–8	–52	70	Left	Postcentral Gyrus	7
	6.4	0	–44	72	Left	Postcentral Gyrus	5
162	7.5	24	–72	26	Right	Precuneus	31
	6.52	30	–62	24	Right	Middle Temporal Gyrus	39
	5.5	30	–62	34	Right	Angular Gyrus	39
69	7.15	–12	–70	–50	Left	Cerebellum	
	5.39	–4	–68	–52	Left	Cerebellum	
133	6.88	–24	–4	0	Left	Putamen	
	6.49	–16	–6	–4	Left	Globus Pallidus	
82	6.78	–28	–18	–14	Left	Hippocampus	
78	6.38	–46	–72	2	Left	Middle Occipital Gyrus	37
	5.96	–42	–66	8	Left	Middle Temporal Gyrus	37

*Coordinates of peak activations (height threshold *p* < 0.05 FWE-corrected; extent threshold *k* = 50 voxels), reported according to the standard atlas of the Montreal Neurological Institute (MNI).*

## Discussion

The present study describes the development and validation of a new methodology that enables to acquire the kinematics of handwriting movements during fMRI studies by means of an innovative MR-compatible tablet. In the literature few studies analyzed the kinematics of handwriting and the related neural correlates (Katanoda et al., 2001; Siebner et al., 2002; Reithler et al., 2006; Reitz et al., 2013; Karimpoor et al., 2015), and none of them raised the question concerning the reliability of the behavioral measurement obtained inside the magnetic bore. This study was motivated by the need to find a methodology that simultaneously allows the acquisition of movement kinematics and brain activity, in order to acquire information representative of the individual’s handwriting features when she/he is in ecological conditions.

Experiment 1 assessed the reproducibility of the kinematic data in two sessions performed one-month apart, giving information concerning the reliability of the system. The results of the acquisitions showed that the kinematic parameters characterizing the sentence the first time the subjects wrote on the tablet did not significantly differ from those acquired after 1 month. Indeed, although a tendency toward the decrease of movement duration and amplitude could be found 1 month later, the existence of significant and positive linear relationships between the first and the second testing times, whose slope values correspond to a moderate-to-strong correlation, suggests that the system is able to provide repeatable and reliable data, as also confirmed by the significant ICC values.

Experiment 2 was performed to test whether and how the kinematic parameters of the sentence were modified when the subjects performed the writing test inside the magnetic bore. Indeed, several factor could potentially affect the measurements and invalidate the test. Firstly, the posture of the subjects was dramatically different from a natural writing posture; the subjects laid down inside the scanner and the tablet was positioned at the level of the pelvis. Some studies specifically focused on evaluating how different body postures influence handwriting movements, producing contrasting results. Although someone did not find any significant tendency regarding posture variation in participants’ signature (Thiery et al., 2013), others reported that some, but not all, writing movement features changed when the subjects assumed a non-conventional writing body posture (Equey et al., 2008; Sciacca et al., 2008, 2011; Dziedzic, 2016). Further, although it is known that movements, and also handwriting movements, obey to some invariant laws of motion (Fitts, 1954; Morasso, 1981; Longstaff and Heath, 1997), writing movements require a precise control of the arm, wrist, hand and fingers that could be severely compromised due to the constrains imposed by the MR environment (the narrow space inside the MR and the request posed to the subject to keep the rest of the body and the head as fixed as possible). Therefore, all these considerations made it difficult to reject a possible influence of the MR environment body posture on writing movement kinematics. Another source of difficulty for the subjects was the impossibility to directly observe their own hand moving, and consequently producing the written trace, as it happens in a natural writing context. Indeed, the possibility to see the hand movement while writing might trigger those eye-hand coordination processes that would be activated during writing in ecological conditions (Gowen and Miall, 2006) and that were proposed to be positively correlated to the quality of handwriting (Kaiser et al., 2009). Even though it was also suggested that visual control is not strictly required to produce automated handwriting movements with respect to proprioception (Marquardt et al., 1996; Hepp-Reymond et al., 2009), in order to mimic as much as possible a natural writing situation, we gave to the subjects the simultaneous visual feedback of what they were writing by means of a custom-made software that allows the contemporary acquisition and display of the trace.

As expected, the results of the comparisons between the kinematic parameters obtained in a writing ecological condition and when the subjects laid down inside the MR scanner showed significant differences; the total movement duration, as well as the height and the length of the sentence significantly increased inside the MR bore. On the other hand, for each parameter, the analysis of the relationship between the acquisition sessions INSIDE and OUTSIDE showed weak-to-moderate, but nevertheless significant correlations, as well as significant ICC values. Therefore, even if the slopes values do not approach the identity (i.e., which corresponds to the perfect reproduction of the data), these findings suggest that the individual features that characterize the subject’s movement with respect to the other participants were sufficiently preserved in the MR environment despite the previously mentioned difficulties. Namely, a subject who was slower than others in ecological writing condition was slower during fMRI, too. Therefore, although with some obvious differences, the kinematic evaluation of writing movements acquired inside the MR scanner can be considered sufficiently descriptive of the individual motor behavior and of the individual differences among subjects. Although this methodology is informative about the temporal and spatial features of handwriting, this study was limited to the description of these kinematic features of writing movement, without assessing differences in the morphology and topology of the letters. This limitation could be considered in future studies that, applying the methodology here proposed, will provide a more comprehensive description of the handwriting outcome.

During the fMRI study, the task required an overt movement execution, which involved hand and fingers’ movements (the forearm was positioned over an MR-compatible support to minimize the subject’s forearm movements); however, participants were able to maintain the head stable and this guaranteed to reduce motion artifacts. No image artifacts due to the presence of the MR-compatible tablet were observed. Further, the fMRI analysis presented here for a representative subject showed the significant activations of brain regions which have been previously described as candidate cortical sites for writing, i.e., the posterior part of the left middle frontal gyrus and the left superior parietal lobule (Vernea and Merory, 1975; Basso et al., 1978), together with the right cerebellum (Katanoda et al., 2001; Purcell et al., 2011; Planton et al., 2013). Further, bilateral activations in the motor areas were observed in line with a previous EEG study (Rupasov et al., 2012). Therefore, one could accept this protocol as effective in engaging the neural networks involved in handwriting tasks.

## Conclusion

In the present study we proposed a combined approach that allows evaluating at the same time the kinematic parameters of handwriting movements and the related brain activity. The system has proven capable of providing reliable kinematic data. Then, despite the expected modifications with respect to an ecological writing movement condition, the kinematic parameters acquired inside the MR scanner were largely descriptive of individuals’ movement features. Further, fMRI results indicate that the cerebral regions activated in this condition were those expected to be involved in an handwriting task in healthy people. Following all these findings, we suggest that this methodology can be promising to evaluate the behavioral performance and simultaneously the brain activations in persons with handwriting difficulties, and provides also a tool to quantitatively evaluate the effects of a rehabilitation treatment. Further studies are also needed to investigate the correlations between the handwriting kinematic parameters and the imaging data in a large number of healthy participants and people with handwriting deficits, where abnormal brain activations might correspond to altered motor behavior.

## Author Contributions

AB conceived and designed the work, performed the acquisition, analyzed and interpreted the data, wrote the manuscript. LP performed the acquisition, analyzed and interpreted the data, revised the manuscript. LB conceived and designed the work, analyzed the data, revised the manuscript. PR interpreted the data, drafted and revised the manuscript, gave the final approval of the version to be published. GB interpreted the data, drafted and revised the manuscript, gave the final approval of the version to be published. MB conceived and designed the work, interpreted the data, revised the manuscript, gave the final approval of the version to be published.

## Conflict of Interest Statement

The authors declare that the research was conducted in the absence of any commercial or financial relationships that could be construed as a potential conflict of interest.
